# Genetic structure of *Miscanthus sinensis* and *Miscanthus sacchariflorus* in Japan indicates a gradient of bidirectional but asymmetric introgression

**DOI:** 10.1093/jxb/eru511

**Published:** 2015-01-24

**Authors:** Lindsay V. Clark, J. Ryan Stewart, Aya Nishiwaki, Yo Toma, Jens Bonderup Kjeldsen, Uffe Jørgensen, Hua Zhao, Junhua Peng, Ji Hye Yoo, Kweon Heo, Chang Yeon Yu, Toshihiko Yamada, Erik J. Sacks

**Affiliations:** ^1^Department of Crop Sciences, University of Illinois, Urbana-Champaign, Urbana, IL 61801, USA; ^2^Department of Plant and Wildlife Sciences, Brigham Young University, Provo, UT 84602, USA; ^3^Field Science Center, Faculty of Agriculture, University of Miyazaki, Miyazaki, Miyazaki 889-2192, Japan; ^4^Faculty of Agriculture, Ehime University, Matsuyama, Ehime 790-8566, Japan; ^5^Department of Agroecology, Aarhus University, Tjele DK-8830, Denmark; ^6^College of Horticulture and Forestry Science, Huazhong Agricultural University, Wuhan, Hubei 430070, China; ^7^Science and Technology Center, China Seed Group Co. Ltd, Wuhan, Hubei 430040, China; ^8^Kangwon National University, Chuncheon, Gangwon 200-701, South Korea; ^9^Field Science Center for Northern Biosphere, Hokkaido University, Sapporo, Hokkaido 060-0810, Japan

**Keywords:** Biomass crop, cross-ploidy introgression, hybridization, Poaceae, polyploidy, population genetics, RAD-seq.

## Abstract

Unilateral introgression from diploids to tetraploids has been hypothesized to be an important evolutionary mechanism in plants. However, few examples have been definitively identified, perhaps because data of sufficient depth and breadth were difficult to obtain before the advent of affordable high-density genotyping. Throughout Japan, tetraploid *Miscanthus sacchariflorus* and diploid *Miscanthus sinensis* are common, and occasionally hybridize. In this study, 667 *M. sinensis* and 78 *M. sacchariflorus* genotypes from Japan were characterized using 20 704 SNPs and ten plastid microsatellites. Similarity of SNP genotypes between diploid and tetraploid *M. sacchariflorus* indicated that the tetraploids originated through autopolyploidy. Structure analysis indicated a gradient of introgression from diploid *M. sinensis* into tetraploid *M. sacchariflorus* throughout Japan; most tetraploids had some *M. sinensis* DNA. Among phenotypically *M. sacchariflorus* tetraploids, *M. sinensis* ancestry averaged 7% and ranged from 1–39%, with introgression greatest in southern Japan. Unexpectedly, rare (~1%) diploid *M. sinensis* individuals from northern Japan were found with 6–27% *M. sacchariflorus* ancestry. Population structure of *M. sinensis* in Japan included three groups, and was driven primarily by distance, and secondarily by geographic barriers such as mountains and straits. *Miscanthus* speciation is a complex and dynamic process. In contrast to limited introgression between diploid *M. sacchariflorus* and *M. sinensis* in northern China, selection for adaptation to a moderate maritime climate probably favoured cross-ploidy introgressants in southern Japan. These results will help guide the selection of *Miscanthus* accessions for the breeding of biomass cultivars.

## Summary

Using high-density genetic markers, gene flow is identified from diploid *Miscanthus sinensis* to tetraploid *M. sacchariflorus* in Japan, in contrast to genetic isolation between these species in China.

## Introduction

High-resolution analyses of population structure, which have been enabled by second-generation sequencing technologies, can provide new insights into the processes of speciation in plants and facilitate crop improvement by guiding marker-trait association studies and identifying groups to test for heterotic combinations. Polyploidy is a primary driver of evolution in flowering plants (Adams and Wendel, 2005; Barabaschi *et al.*, 2012), and it has long been recognized that polyploidization of amphidiploids can result in new, genetically isolated species in a single event (Winge, 1917; Stebbins, 1959). Additionally, introgression between plant species plays an important role in local adaptation and speciation (Rieseberg *et al.*, 2003; Arnold, 2004; Arnold *et al.*, 2008). In contrast to the genetic isolation typically observed between populations of different ploidies, Stebbins (1971) noted that introgression across ploidy levels can also occur in plants. Moreover, Stebbins (1971) postulated that introgression of genes between diploid and tetraploid populations would usually flow preferentially from diploids to tetraploids (via unreduced gametes and/or triploid bridges) and these gene movements could have large evolutionary consequences (Petit *et al.*, 1999). To date, however, few examples of gene flow from diploids to tetraploids have been reported for wild plants (Kim *et al.*, 2008; Wang *et al.*, 2014), but prior technological limitations in the ability to detect small introgressions in a large sampling of genotypes and populations may have contributed to a lack of reports. The extent to which polyploidy has contributed to speciation or, in contrast, limited differentiation of populations via gene exchange, can now be explored in detail with analyses of population structure using high marker densities.

*Miscanthus* is a genus of perennial grasses native to east Asia and Oceania, and includes polyploid and diploid species that are able to hybridize. A close relative of sugarcane (*Saccharum* hybrids), *Miscanthus* is also useful in its own right as a lignocellulosic biomass crop, and as a popular ornamental in European and North American gardens. However, there has been little or no effort to domesticate *Miscanthus* in its native lands. *M. sacchariflorus* (Maxim.) Hack. and *M. sinensis* Andersson are among the most widely distributed and divergent species within *Miscanthus sensu stricto* and are the parent species of the biomass crop *M.*×*giganteus* (Hodkinson *et al*., 2002*a*,*b*; Clifton-Brown *et al.*, 2008; Sacks *et al.*, 2013). *M. sinensis* has a caespitose form, prefers aerobic soils especially on hilly sites that are infrequently disturbed by grazing or fire, and is typically diploid with a monoploid genome size of about 2.5–2.8 pg (Clifton-Brown *et al.*, 2008; Rayburn *et al.*, 2009; Sacks *et al.*, 2013; Li *et al.*, 2013; Jiang *et al.*, 2013; Moon *et al.*, 2013; Chae *et al.*, 2014). In contrast, *M. sacchariflorus* has a spreading rhizomatous habit, prefers riparian environments, and can be diploid or tetraploid with a monoploid genome size of about 2.1–2.3 pg (Rayburn *et al.*, 2009; Li *et al.*, 2013; Moon *et al.*, 2013; Chae *et al.*, 2014). Though the natural ranges of *M. sinensis* and *M. sacchariflorus* overlap from ~29° N to 43° N, *M. sinensis* is distributed further south to at least ~18° N in Hainan, whereas *M. sacchariflorus* is distributed further north to ~50° N in eastern Russia (Sacks *et al.*, 2013). Thus, *M. sinensis* and *M. sacchariflorus* are well differentiated phylogenetically, morphologically, and ecologically.

Throughout Japan, diploid *M. sinensis* and tetraploid *M. sacchariflorus* are common, and although they typically occupy different niches, sympatric populations occur. Though both diploid and tetraploid *M. sacchariflorus* have been found in mainland Asia (Yan *et al.*, 2012; Li *et al.*, 2013; Moon *et al.*, 2013), an extensive survey in Japan reported only tetraploids (Hirayoshi *et al.* 1957).

Hodkinson and Renvoize (2001) defined the nothospecies *M.*×*giganteus* J.M. Greef and Deuter ex Hodkinson and Renvoize (syn. *M. ogiformis* Honda if awns present; Honda, 1939) as a hybrid between *M. sacchariflorus* and *M. sinensis*. In 1935, a single triploid *M.*×*giganteus* genotype was exported from Japan to Denmark (Greef *et al.*, 1997; Głowacka *et al.*, 2014). This *M.*×*giganteus* genotype has become an important crop for the emerging lignocellulosic bioenergy industry in Europe and North America owing to its high yield, low input requirements, low risk of invasiveness, high rate of photosynthesis at low temperatures, and broad adaptation (Barney and Ditomaso, 2008; Pyter *et al.*, 2009; Somerville *et al.*, 2010; Purdy *et al.*, 2013). Subsequent to its initial introduction to Europe, a few additional triploid *M.*×*giganteus* genotypes have been found growing *in situ* in Japan (Adati and Shiotani, 1962), and others have been obtained by germinating seed collected from wild plants of *M. sinensis* (Hirayoshi *et al.*, 1957) or *M. sacchariflorus* (Nishiwaki *et al.*, 2011; Dwiyanti *et al.*, 2013) from locations where both species grew sympatrically.

In mainland Asia, where diploid *M. sacchariflorus* is common, it naturally crosses with *M. sinensis* to produce homoploid hybrids that have previously been named *M. purpurascens* or *M. sinensis* var. *purpurascens* (Jiang *et al.*, 2013; Chae *et al.*, 2014; Głowacka *et al.*, 2014). These diploid interspecific hybrids backcross infrequently with *M. sinensis* but do not form a hybrid swarm (Jiang *et al.*, 2013; Clark *et al.*, 2014). However, the extent of genetic exchange between *M. sinensis* and *M. sacchariflorus* in Japan, where *M. sacchariflorus* is thought to be exclusively tetraploid, is unknown beyond the occasional production of sterile triploid *M.*×*giganteus* hybrids (Hirayoshi *et al.*, 1957; Adati and Shiotani, 1962).

In addition to the discovery of new *M.*×*giganteus* genotypes in nature, human-directed crosses between diploid *M. sinensis* and tetraploid *M. sacchariflorus* can be made intentionally, utilizing germplasm with desired traits and exploiting the genetic diversity of these obligate-outcrossing species to maximize heterosis. Previous efforts to breed new triploid genotypes of *M.*×*giganteus* by Hirayoshi *et al.* (1960), and the release of ‘Nagara’ in 2006 by M. Deuter of Tinplant (Klein Wanzleben, Germany) indicate that this approach is viable. Recently, more than 30 new triploid *M.*×*giganteus* genotypes have been bred at the University of Illinois and field evaluations of these have begun. Crucial to the success of breeding new biomass cultivars of *M.*×*giganteus* will be an in-depth understanding of genetic diversity and population structure for *M. sinensis* and *M. sacchariflorus* to guide the selection of parental genotypes for combining ability, adaptation, and novel alleles.

In a previous study, a broad survey of *M. sinensis* genetic diversity with accessions primarily from China, Korea and Japan was conducted, and six groups were identified, including one each in northern Japan (northern Honshu and Hokkaido) and southern Japan (Clark *et al.*, 2014). It was also found that nearly all of the ornamental cultivars of *M. sinensis* grown in the USA were derived from southern Japan. However, there has not yet been a population genetic study with sufficient resolution to observe how the genetic structure of *M. sinensis* in Japan was affected by geographic features such as straits and mountain ranges. A limitation of the previous study (Clark *et al.*, 2014) was that only 131 wild-collected *M. sinensis* genotypes from Japan were able to be evaluated, with only 34 of those from central and southern Japan, and no Japanese *M. sacchariflorus*. Thus, the current study was conducted to provide an in-depth understanding of *M. sinensis* population structure in Japan as it relates to geography, and to establish a baseline understanding of *M. sacchariflorus* diversity in Japan, whereas the previous study was a broad East Asia-wide assessment of relationships among *M. sinensis* populations. Very little is known about the genetic structure of *M. sacchariflorus*, including the relationship between its diploid and tetraploid forms, and the amount of introgression with *M. sinensis*, if any. Indeed, there is longstanding disagreement about whether tetraploid *M. sacchariflorus* is allo-, segmental-, or auto-polyploid (Adati, 1958, 1959; Adati and Shiotani, 1962; Takahashi and Shibata, 2002; Chae *et al.*, 2014). Thus, the objectives of this study were to (i) detect spatial genetic structure of Japanese *M. sinensis* and *M. sacchariflorus*, (ii) more precisely identify the genetic origins within Japan of ornamental and US naturalized *M. sinensis*, and (iii) assess the degree of hybridization and introgression between *M. sinensis* and *M. sacchariflorus* in Japan, and determine the ploidy of any hybrids.

## Materials and methods

### Plant materials and genotyping

In total, 1513 genotypes of *Miscanthus* were studied. Focus was especially placed on 667 *M. sinensis* genotypes from 202 accessions, and 78 *M. sacchariflorus* genotypes from 53 accessions, collected from the wild in Japan and studied for the first time here (i.e. the Japan dense-sampling set; Table 1, Supplementary dataset S1). Germplasm from the Japan dense-sampling set was collected as seed and/or clonal propagules in 1996 and from 2007–2011. Each seed accession was a bulk collection from between one and 50 mother plants, whereas each clonal accession came from a single individual. In addition to the 255 accessions (745 individuals) from the Japan dense-sampling set, we also studied 622 *M. sinensis* and four *M. floridulus* (Labill.) Warb. ex K. Schum. & Lauterb. accessions (one genotype per accession) primarily from China, Korea, and Japan, 11 *M. sacchariflorus* from China and Korea, and eight *M. sinensis* × *M. sacchariflorus* F1 hybrids from China that we evaluated previously (i.e. the region-wide set; Supplementary dataset S1; Clark *et al.,* 2014), in order to understand relationships among accessions from Japan in a regional context. The Japanese *Miscanthus* accessions were also compared to 79 diploid *M. sinensis* or *M. sinensis*×*M. sacchariflorus* ornamental cultivars available in the USA, 42 naturalized *M. sinensis* genotypes from 13 accessions collected in the USA, one diploid *M. sacchariflorus* ornamental cultivar, and the triploid biomass cultivar *M.*×*giganteus* ‘Illinois’ (Supplementary dataset S1).

**Table 1.** *Origins of* Miscanthus *accessions genotyped in the present study*

Island	Species	Seed only	Clonal only	Clonal + seed
Hokkaido	*M. sinensis*	91		
	*M. sacchariflorus*	2	3	
Honshu	*M. sinensis*	88	3	
	*M. sacchariflorus*	3	32	
Shikoku	*M. sinensis*	5		
Kyushu	*M. sinensis*	14	1	
	*M. sacchariflorus*	1	6	
Total	*M. sinensis*	198	4	6
	*M. sacchariflorus*	6	41	6

Restriction site-associated DNA sequencing (RAD-seq) and plastid genotyping were performed using methods described previously (Clark *et al.*, 2014). For RAD-seq genotyping, a *Pst*I–*Msp*I digestion was used to sequence tags adjacent to *Pst*I sites, and 95 barcoded samples were multiplexed into each of ten libraries. Each library was run in one lane on a HiSeq 2000 (Illumina, San Diego, California, USA) for 100bp single-end reads at the University of Illinois Roy J. Carver Biotechnology Center DNA Sequencing Unit. All sequencing data has been deposited in the NCBI Sequence Read Archive, BioProject ID PRJNA261699. All samples were also genotyped with ten plastid microsatellite markers (de Cesare *et al*., 2010; Jiang *et al.*, 2012) scored by electrophoresis on an ABI 3730 (Applied Biosystems, now part of Thermo Fisher Scientific, Waltham, Massachusetts, USA) followed by allele calling in STRand (Toonen and Hughes, 2001).

### Genetic data analysis

The UNEAK pipeline in TASSEL 3.0.162 (Lu *et al.*, 2013) was used to call single nucleotide polymorphism (SNP) genotypes from RAD-seq data using a minimum call rate of 0.5 and a minimum minor allele frequency of 0.01. In addition to the 745 individuals from the Japan dense-sampling set, 645 individuals from the region-wide set, 42 US naturalized genotypes, and 81 cultivars (Clark *et al.*, 2014) were included in the SNP-calling pipeline, yielding 20 704 SNPs after removing SNPs that appeared heterozygous in one or more doubled haploid lines. Though polyploidy represents a challenge for SNP-calling, the UNEAK pipeline was designed to distinguish paralogues in polyploids and the use of doubled haploid *M. sinensis* lines further enabled this differentiation (Clark *et al.*, 2014).

SNPs were analysed with the software Structure 2.3.4 (Falush *et al.*, 2003) to identify new genetic groups, assign individuals to previously identified groups (Clark *et al.*, 2014), and detect admixture and hybridization between species (see also Supplementary Materials and methods). Structure Harvester (Earl and VonHoldt, 2011) was used to determine the best number of clusters (K). To determine the origins of ornamental and naturalized accessions of *M. sinensis* available in the US, the USEPOPINFO and PFROMPOPFLAGONLY options were used. To determine the power of Structure to detect hybridization, analyses were conducted on groups of simulated hybrid individuals using individuals from the dataset as parents, and on a simulated population of individuals from the common ancestor of *M. sinensis* and *M. sacchariflorus*. Principal components analysis performed with adegenet (Jombart and Ahmed, 2011) was also used to compare and validate the results from Structure.

The R package mmod (Winter, 2012) was used to calculate the differentiation statistic Jost’s *D* (Jost, 2008) using the 20 704 RAD-seq SNPs between pairs of groups as identified by discriminant analysis of principal components (DAPC; Jombart *et al.*, 2010). Jost’s *D* was calculated individually for each locus, then averaged across loci. Nei’s *D* (expected heterozygosity) was calculated for the same groups using allele frequencies calculated by the *glMean* function in adegenet (Jombart and Ahmed, 2011). To control for differences in group size, for each genetic group, 500 jack-knifed subgroups containing 100 individuals each were used to calculate Nei’s *D*, and the mean and standard error were calculated across jack-knifed replicates. *F*_ST_ was calculated in the R package pegas (Paradis, 2010) to determine the differentiation of each Japanese *M. sinensis* genetic group from Japanese *M. sinensis* as a whole.

Spatial principal components analysis (sPCA), implemented in the R package adegenet (Jombart *et al.*, 2008) was used to identify spatial patterns in genetic variation of *M. sinensis* across the major islands of Japan using RAD-seq SNPs. The R package ade4 (Chessel *et al.*, 2004) was used to plot the results.

A haplotype network was generated from all ten chloroplast microsatellite markers, as in Clark *et al.* (2014). Any individuals with missing data were removed from the haplotype network analysis, leaving 731 *M. sinensis* and *M. sacchariflorus* individuals from 252 accessions from the Japan dense-sampling set.

### Flow cytometry

Flow cytometry was performed using a protocol modified from Rayburn *et al.* (2009). Flow cytometry was used to determine the nuclear DNA content of all *M. sacchariflorus* and *M.*×*giganteus* individuals for which live plants were available (72 out of 78 from the Japan dense-sampling set, plus two from Korea and nine from China from the region-wide set), as well as a sample of 32 *M. sinensis* individuals from the Japan dense-sampling set.

## Results

### Major groupings, admixture, and hybridization of *Miscanthus* based on SNP data

**Fig. 1.**
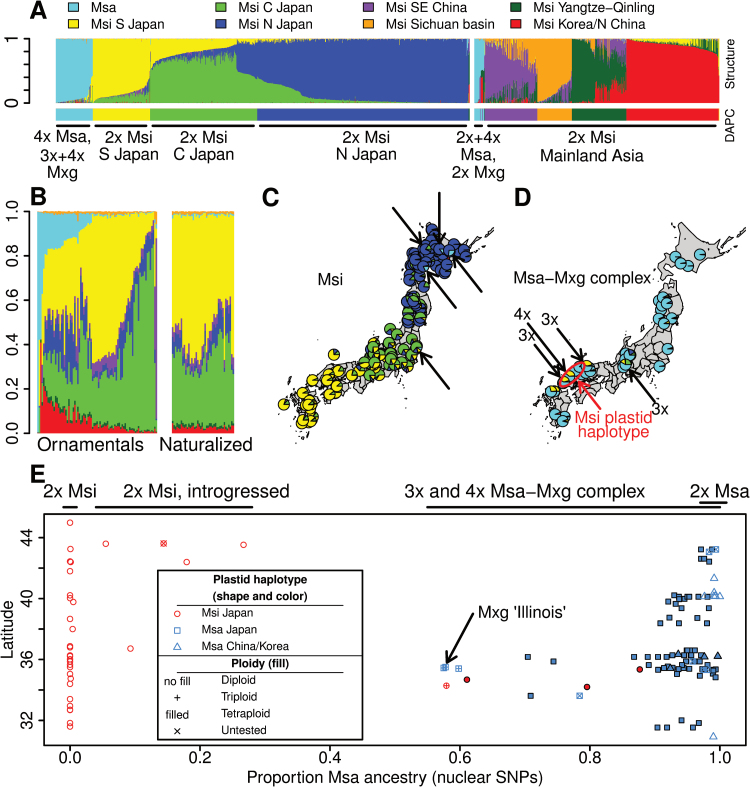
Structure and DAPC results using 20 704 nuclear SNPs. Msi=*Miscanthus sinensis*, Msa=*M. sacchariflorus*, Mxg=*M.*×*giganteus*. (A) Bar plot of Q values (proportion ancestry estimated in Structure) for 745 individuals from the Japan dense-sampling set and 645 individuals from the previously published region-wide set (Clark *et al.*, 2014). Each of five runs included 253 individuals from the Japan-dense set (one per accession) plus all 645 individuals from the region-wide set; mean Q values are shown for individuals that were present in more than one run. Each of the eight groups is represented by a different colour. The narrower bottom bar indicates DAPC group assignments. (B) Mean Q values for 81 ornamental individuals and 42 naturalized individuals from the USA, when the parameters USEPOPINFO and PFROMPOPFLAGONLY were used in Structure to assign ancestry from native populations. (C) Map of Q values for Msi individuals in Japan, including 667 from the Japan-dense set and 128 from the region-wide set. Five individuals with Msa ancestry 6–27%, including four diploids and one of undetermined ploidy, are indicated with arrows. (D) Map of Q values for 78 Msa–Mxg complex individuals from Japan, all from the Japan dense-sampling set. Four individuals with Msi ancestry 39–42% are indicated with arrows, and the ploidy determined by flow cytometry is indicated; all other individuals shown were tetraploid except for six of undetermined ploidy. The red ellipse indicates the sampling area for all four Msa–Mxg individuals with an Msi plastid haplotype (other individuals within the ellipse have an Msa plastid haplotype). (E) Latitude vs Q values for 89 native-collected Msa–Mxg complex individuals, Mxg ‘Illinois’ (assuming origin in Yokohama, Japan; indicated with an arrow), 28 random Msi individuals that were subjected to flow cytometry, and five Msi individuals with Msa ancestry >5%. Colour and shape of symbols in (E) are used redundantly to indicate plastid haplotype and collection location, and fill is used to indicate ploidy, with filled points outlined in black to make them more easily visible.

Structure analysis of the Japan dense-sampling set identified K=4 (three *M. sinensis* and one *M. sacchariflorus*) as the most reproducible estimate (Supplementary Fig. S1). Thus, the high density sampling in this study enabled identification of three *M. sinensis* groups in Japan (northern, central, and southern, hereafter called N, Central, and S Japan when referring to genetic clusters as opposed to geographic regions; Fig. 1A, C), where previous low density sampling had identified only two groups (northern and southern). A combined analysis of the Japan dense-sampling set with the region-wide set at K=8 identified the seven genetic groups from the previous study (six *M. sinensis* and one *M. sacchariflorus*; Clark *et al.*, 2014) plus the one additional *M. sinensis* group identified in the analysis of the Japan dense-sampling set (Fig. 1A, C). The first principal component of the SNP data was strongly correlated with *M. sacchariflorus* ancestry identified by Structure (*r* ^2^=0.99; Supplementary Fig. S2A), and Structure runs on simulated hybrids indicated that even highly backcrossed (BC_5_) individuals could be distinguished from the parent species (Supplementary Fig. S2B, Table S1).

**Fig. 2.**
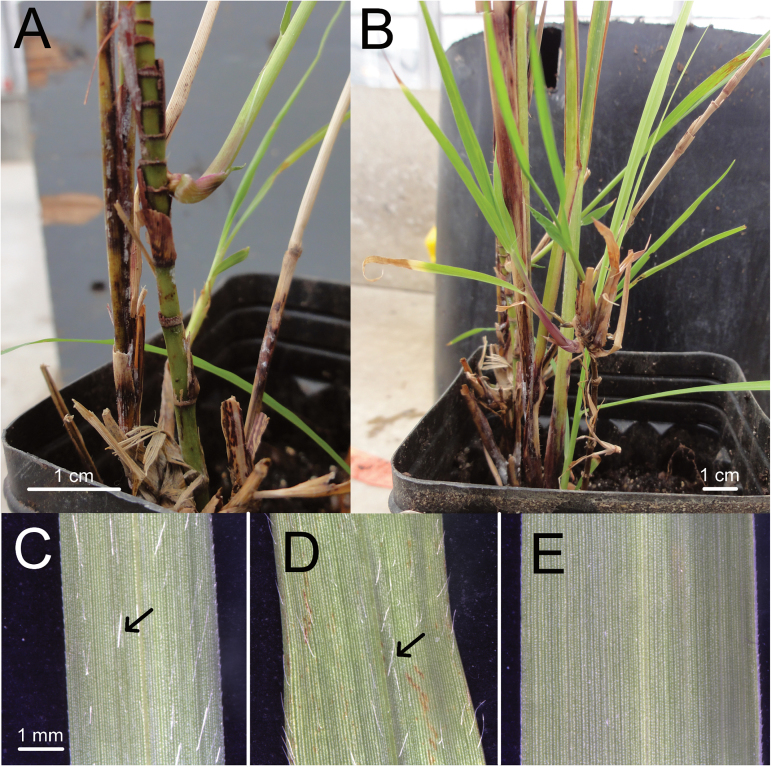
Photographs of EBI-2009-02c, an *M. sinensis*×*M. sacchariflorus* individual grown from seed collected in Hokkaido, Japan. Ancestry of EBI-2009-02c according to Structure was ~73% *M. sinensis* from N Japan and ~27% *M. sacchariflorus* (Fig. 1A, C, E). Its plastid haplotype was commonly found among *M. sinensis* in Hokkaido (haplotype C, Fig. 4). (A) Close-up showing axillary branching and long internodes, which are characteristic of *M. sacchariflorus*. (B) Broader view, with more branching visible. (C–E) Abaxial leaf surface of three plants, showing presence or absence of trichomes. Scale is identical in C–E. (C) Non-hybrid *M. sinensis* displaying trichomes (arrow), which is typical for this species. (D) EBI-2009-02c, with trichomes (arrow). (E) Diploid non-hybrid *M. sacchariflorus*, with a glabrous phenotype that is typical of this species.

Based on admixture estimates, *M. sinensis* genotypes in Japan were strongly isolated from each of the other five groups identified (Fig. 1A, Supplementary Dataset S1). Isolation of *M. sinensis* from *M. sacchariflorus* in Japan was especially strong. Only 9 of the 667 phenotypically *M. sinensis* genotypes evaluated had <99% *M. sinensis* ancestry. Unexpectedly, however, four diploid individuals from Hokkaido and one from Ibaraki (central Honshu) had hybrid ancestry >5% from *M. sacchariflorus* (27%, 18%, 14%, 6%, and 9% respectively), and were part of seed accessions that were otherwise non-hybrid (EBI-2009-02c, Koike-05a, EBI-2008-46c, EBI-2008-37e, JA55-2c; Supplementary dataset S1). For EBI-2009-02c, intermediate morphological characteristics were observed between *M. sacchariflorus* and *M. sinensis*, including axillary branching, which is characteristic of *M. sacchariflorus*, and trichomes on the abaxial surface of leaves, which is characteristic of *M. sinensis* (Fig. 2). Among the Japanese genotypes with ≥99% *M. sinensis* ancestry, only 39 out of 795 had less than 95% Japanese ancestry. Most of the non-Japanese admixture observed for *M. sinensis* from Japan was with the southeast (SE) China *M. sinensis* group (Fig. 1A, Supplementary Dataset S1).

Of the three Japanese *M. sinensis* genetic groups (as identified by DAPC), the N Japan group was the least diverse and the Central Japan group was the most diverse, both in terms of plastid haplotypes and nuclear SNPs (Table 2). Among the three Japanese *M. sinensis* groups, S Japan was the most differentiated from the others based on *F*_ST,_ and Central Japan was the least differentiated (Table 2). Pairwise Jost’s *D* between DAPC groups revealed that the S Japan group was more closely related to the SE China group than to the N Japan group (Table 3). Of the three *M. sinensis* groups in Japan, S Japan was the most closely related to each mainland Asia group, and N Japan was the most distantly related (Table 3).

**Table 2.** *Diversity statistics for Japanese groups of* M. sinensis *(Msi) and the* M. sacchariflorus–M.×giganteus *complex (Msa–Mxg)* For Msi, the Gini-Simpson index and Nei’s *D* were estimated using 500 jack-knifed groups containing 100 individuals from each Msi group, and standard errors are calculated across jack-knifed replicates. For Msa–Mxg, the Gini-Simpson index was estimated without jack-knifing owing to small sample size, and Nei’s *D* was not calculated because of expected bias from the SNP-mining method (most individuals used for SNP mining in UNEAK were Msi). *F*_ST_ indicates differentiation of each Japanese Msi group from the other two, and the standard error is given across loci.

Group	Number of individuals	Number of plastid haplotypes	Gini-Simpson index, plastid haplotypes	Nei’s *D*, nuclear SNPs	*F*_ST_, nuclear SNPs
N Japan Msi	446	16	0.4533±0.0022	0.13055±0.00003	0.0421±0.0004
C Japan Msi	226	19	0.7747±0.0011	0.13460±0.00002	0.0238±0.0003
S Japan Msi	122	17	0.7606±0.0002	0.13217±0.00001	0.0455±0.0005
All Japan Msi	794	38	0.7911±0.0006	0.14247±0.00001	
Japan Msa–Mxg	78	19	0.8340±0.0375		

**Table 3.** *Pairwise Jost’s D of Japanese* M. sinensis *groups* Mean and standard error were calculated across 20 704 SNP loci. Colour names correspond to colours in Fig. 1.

	C Japan	S Japan	Korea, N China (red)	SE China (purple)	Yangtze-Qinling (dark green)	Sichuan (orange)	Msa (cyan)
N Japan (blue)	0.0169±0.0003	0.0313 ± 0.0006	0.0571±0.0009	0.0492±0.0008	0.0597±0.0009	0.0743±0.0011	0.1224±0.0017
C Japan (light green)		0.0127±0.0003	0.0403±0.0007	0.0326±0.0006	0.0435±0.0008	0.0592±0.0010	0.1080±0.0016
S Japan (yellow)			0.0337±0.0006	0.0294±0.0006	0.0395±0.0007	0.0567±0.0010	0.1058±0.0016

Ornamental *M. sinensis* and *M. sinensis*×*M. sacchariflorus* accessions from the USA had, on average, 39% ancestry to the S Japan genetic group, 32% to the Central Japan group, 11% to the N Japan group, 6% to the Korea/N China group, and 7% to the *M. sacchariflorus* group (Fig. 1B). Naturalized *M. sinensis* accessions collected in the USA had more uniform Q values among individuals than ornamental cultivars, and most of their ancestry was from S and Central Japan. Although *M. sacchariflorus* ancestry was negligible (0.7%) within the naturalized USA accessions, they did have 2.6% ancestry from the Korea/N China group, whereas native Japanese *M. sinensis* only had 0.2% ancestry from the Korea/N China group.

For all *M. sacchariflorus* studied, including those from Japan, China and Korea, a single group was identified via Structure analysis (Fig 1A). Nine individuals from S Japan with *M. sacchariflorus* phenotypes (six of which were collected as clones and three as seeds) had hybrid ancestry (Q values) >20% from *M. sinensis*, three of which were triploid and five tetraploid (ploidy was undetermined for one individual owing to loss of the plant; Fig. 1A, D, E, Table 4); thus, these individuals were probably F_1_ and BC_1_ interspecific hybrids (i.e. *M.*×*giganteus*). Only 11 of the 69 phenotypically *M. sacchariflorus* Japanese genotypes that were confirmed to be tetraploid had ≥98% of their nuclear alleles from *M. sacchariflorus*, with the remaining accessions having *M. sinensis* ancestry (predominantly Japanese) ranging from 2–39%, with a mean of 7% and median of 5% (Fig. 1A and E). Thus, recurrent backcrossing of hybrid individuals probably produced the observed gradient of *M. sinensis* introgression into *M. sacchariflorus* (Fig. 1E). For phenotypically *M. sacchariflorus* individuals with <20% *M. sinensis* ancestry, latitude was negatively correlated with *M. sinensis* ancestry (Fig. 1E), indicating that introgression was more frequent in southern Japan than northern Japan. In contrast to the frequent introgression of *M. sinensis* genes into tetraploid *M. sacchariflorus* in Japan, seven diploid and three tetraploid *M. sacchariflorus* from China each had ≥98% *M. sacchariflorus* ancestry. However, two *M. sacchariflorus* from Korea had only 92–94% *M. sacchariflorus* ancestry, with most of the remainder from the Korea/N China *M. sinensis* group.

**Table 4.** *F_1_ and BC_1_* M.×giganteus *collected from the wild in Japan* *M.*×*giganteus* ‘Illinois’ is included for comparison. Msa=*M. sacchariflorus*. Proportion Msa ancestry=Q value estimated by Structure.

Accession	Type	Prefecture	Proportion Msa ancestry	Ploidy	Plastid haplotype group
JM11-006	Clone	Yamaguchi	0.796	4×	B
JA52a	Seed	Fukuoka	0.784	NA (dead plant)	Msa
Gifu-2010-020d	Seed	Gifu	0.744	4×	Msa
JM11-002	Clone	Fukuoka	0.709	4×	Msa
Gifu-2010-014a	Seed	Gifu	0.705	4×	Msa
JM11-013	Clone	Shimane	0.611	4×	B
JM11-031	Clone	Tottori	0.598	3×	Msa
JM11-010	Clone	Yamaguchi	0.579	3×	B
Gifu-2010–025	Clone	Gifu	0.578	3×	Msa
‘Illinois’	Clone	Kanagawa	0.575	3×	Msa

### Spatial analysis of *M. sinensis* SNP data

**Fig. 3.**
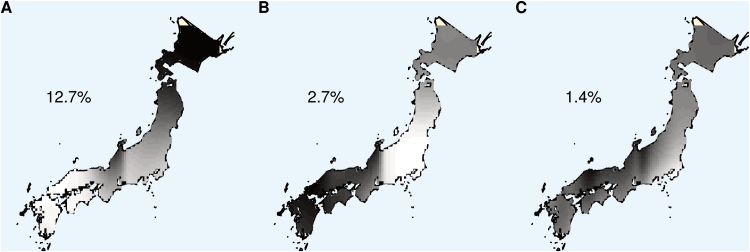
Spatial principal components analysis (sPCA) of *M. sinensis* in Japan using 5359 SNPs across 782 individuals from 205 collection sites. (A–C) Interpolation of scores of lag vectors of the first three eigenvectors produced by sPCA. Scores are represented the darkness of greyscale pixels. The percentage of genetic variation between sites explained by each of the three eigenvectors is indicated. (This figure is available in colour at *JXB* online.)

If *M. sinensis* individuals were sorted by Q value, the values changed abruptly in several regions of the bar plot, suggesting barriers to gene flow (Fig. 1A). Spatial principal components analysis of nuclear SNP data indicated the geographical locations and relative strengths of these barriers to gene flow for *M. sinensis* in Japan (Fig. 3). The first three eigenvectors with positive spatial autocorrelation were chosen for analysis, based on a screeplot of genetic variance vs spatial autocorrelation (Supplementary Fig. S3). The first eigenvector, which had by far the highest variance (Fig. 3A; 12.7% of genetic variation between sites), represented a genetic gradient north to south in Japan, as well as differentiation of the region to the southwest of the Noto Peninsula. The second eigenvector, representing 2.7% of the genetic variation between sites, revealed central Honshu as the most divergent region, and a steep genetic cline near the Japanese Alps (Fig. 3B). The third eigenvector, representing 1.4% of genetic variation between sites, showed a gradient from east to west (Fig. 3C). None of these three eigenvectors revealed genetic structure within Hokkaido despite the large sample size in that region.

### Plastid microsatellites

**Fig. 4.**
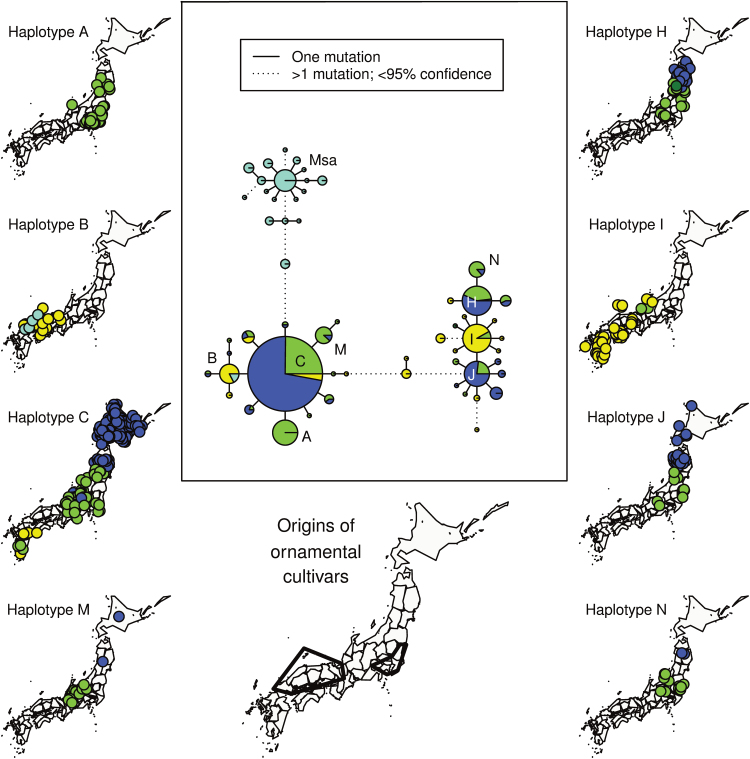
Haplotype network of *Miscanthus* based upon ten plastid microsatellite markers, and maps of sampling locations of the most common haplotypes. Circle area in the network is proportional to the number of unique accessions with each haplotype, and pie slice area is proportional to the number of individuals in each DAPC group as determined by nuclear SNPs (Fig. 1A, lower bar, with corresponding colours). Msa indicates the sub-network belonging to *M. sacchariflorus*. The rest of the network is found in *M. sinensis*. The eight most common haplotypes in *M. sinensis* are indicated with letters. Haplotypes A, B, C, H, I, and J correspond to identically named haplotypes from Clark *et al.* (2014). Probable geographic origins of ornamental *M. sinensis* cultivars (bottom, centre) were determined by the presence of haplotypes A, B, and I and the absence of other haplotypes.

Across the Japan dense-sampling set of 745 individuals (255 accessions), 57 unique plastid haplotypes were identified. From these, a haplotype network was calculated (Fig. 4), which included a sub-network of haplotypes specific to *M. sacchariflorus* and two major sub-networks of haplotypes common in *M. sinensis*. The topology of the haplotype network (Fig. 4) was slightly different from the previously published network (Clark *et al.*, 2014) owing to homoplasy of microsatellite alleles, absence of haplotypes present only in mainland Asia, and/or differences in haplotype frequency. Strong geographic structure was seen among the major *M. sinensis* haplotypes (Fig. 4), including haplotypes A and B which are common among ornamental cultivars available in the USA and Europe (Clark *et al.*, 2014). Of the 78 phenotypically *M. sacchariflorus* individuals (53 accessions) genotyped, four did not have a plastid haplotype that was part of the *M. sacchariflorus* sub-network, but instead had haplotype B, an *M. sinensis* haplotype common in Shikoku and southern Honshu where those four accessions were collected (Figs. 1D, E and 4) and not found anywhere else in Asia (Clark *et al.*, 2014). These four interspecific hybrid individuals with *M. sinensis* plastids were collected along the west coast of Chūgoku (Fig. 1D) and 58–88 % of their nuclear DNA was from *M. sacchariflorus* with the remainder from *M. sinensis*; one individual was triploid and the others were tetraploid. Though *M. sinensis* plastids were found introgressed into *M. sacchariflorus*, *M. sacchariflorus* plastids were not found introgressed into *M. sinensis*.

### DNA content

All tested individuals that had >60% *M. sacchariflorus* ancestry were tetraploid, with the exception of seven diploids from China (Fig. 1E). As expected, all tested *M. sinensis* individuals were diploid (Fig. 1E). Of the five interspecific hybrids that phenotypically resembled *M. sinensis* and had *M. sinensis* plastids, but had 6–27% *M. sacchariflorus* ancestry based on nuclear SNPs, four were determined to be diploid (Fig. 1E), and the fifth individual died before it could be tested.

## Discussion

### Introgression of *M. sinensis* DNA into tetraploid *M. sacchariflorus*

Though previous studies have also identified triploid hybrids between tetraploid *M. sacchariflorus* and diploid *M. sinensis* from wild populations in Japan (Hirayoshi *et al.*, 1957, 1960; Adati and Shiotani, 1962; Hodkinson *et al.*, 2002c; Nishiwaki *et al.*, 2011; Dwiyanti *et al.*, 2013) and even one interspecific tetraploid hybrid (Dwiyanti *et al.*, 2013), this is the first study to establish that introgression of *M. sinensis* DNA into tetraploid *M. sacchariflorus* is common in Japan, resulting in a tetraploid population that has a continuous gradient of *M. sinensis* nuclear genetic ancestry ranging up to 39%. Only 16% of the phenotypically *M. sacchariflorus* tetraploids from Japan had ≥98% *M. sacchariflorus* ancestry, whereas all nine of the *M. sacchariflorus* from China that were studied (seven diploids and two tetraploids) exceeded this threshold. Similar to the unintrogressed *M. sacchariflorus* from China, ≥99% of *M. sinensis* from Japan had ≥99% *M. sinensis* ancestry (Fig. 1A, E). Moreover, the amount of introgression of *M. sinensis* DNA into *M. sacchariflorus* was negatively correlated with latitude (Fig. 1E), and is highest where flowering times of *M. sinensis* and *M. sacchariflorus* have the greatest overlap and where alleles for adaptation to a warm climate would be expected to have the greatest benefit to *M. sacchariflorus*. Thus, the tetraploid *Miscanthus* in Japan, which had been considered to be allo- or autopolyploid *M. sacchariflorus*, are in fact predominantly a hybrid swarm derived from autotetraploid *M. sacchariflorus* and diploid *M. sinensis*. In contrast to the tetraploid hybrid swarm that we identified in Japan, Jiang *et al.* (2013) reported that hybrids between diploid *M. sacchariflorus* and diploid *M. sinensis* in China had approximately equal genetic contributions from both parents, and they did not find evidence of introgression from one species into the other. Another notable contrast between the tetraploid interspecific hybrids in Japan and diploid interspecific hybrids in China is that the tetraploids in Japan are phenotypically most similar to the *M. sacchariflorus* parent, including the development of long rhizomes, whereas the interspecific diploids in China are phenotypically most similar to the *M. sinensis* parent, including caespitose habit. Such differences in the growth characteristics of the hybrids would be expected to have substantial effects on their adaptation and competitiveness.

This study is also the first to report *M. sinensis* plastids introgressed into wild-collected tetraploid *M. sacchariflorus* from Japan, with about 4% of the phenotypically *M. sacchariflorus* tetraploids having *M. sinensis* plastids in nuclear genetic backgrounds that ranged from 61–88% *M. sacchariflorus*. Consistent with the findings of *M. sinensis* plastids introgressed into wild *M. sacchariflorus* tetraploids from Japan, Hirayoshi *et al.* (1960) produced a triploid and a tetraploid progeny from a purposeful cross between diploid *M. sinensis* var. *condensatus* as the female parent and tetraploid *M. sacchariflorus* as the male parent. In China, wild-collected hybrids between diploid *M. sacchariflorus* and diploid *M. sinensis* were also found to have plastids from either parent (Jiang *et al.*, 2013; Clark *et al.*, 2014).

Cytogenetic evidence has resulted in conflicting reports as to whether tetraploid *M. sacchariflorus* is allopolyploid or autopolyploid, although the most modern studies suggest autopolyploidy (Adati and Shiotani, 1962; Takahashi and Shibata, 2002). The present study also does not support the allopolyploid hypothesis. If the *M. sacchariflorus* in Japan were allotetraploid, derived from diploid *M. sacchariflorus* and diploid *M. sinensis*, it would be expected that at least half their ancestry would be from *M. sinensis*, but this was not observed, as *M. sinensis* ancestry was typically <20% and never greater than 39% (Fig 1E). Moreover, it was found that the tetraploid *M. sacchariflorus* from Japan co-clustered with the diploid and tetraploid *M. sacchariflorus* from China and Korea. Thus, tetraploid *M. sacchariflorus* in Japan probably originated via autopolyploidization of diploid *M. sacchariflorus*, but subsequent and ongoing crosses with diploid *M. sinensis* have resulted in a predominantly interspecific hybrid population of tetraploids in Japan. Given that both diploid and tetraploid hybrids between *M. sacchariflorus* and *M. sinensis* are typically fertile and have normal meioses (Hirayoshi *et al.*, 1960; Jiang *et al.*, 2013; Clark *et al.*, 2014), their genomes may not be sufficiently differentiated to result in allopolyploid speciation of hybrids. Whereas polyploidization of amphidiploids is typically considered a speciation event, polyploidization of *M. sacchariflorus* has facilitated considerable introgression of genes from diploid *M. sinensis* in Japan (i.e. brought the nascent differentiating genomes of *M. sacchariflorus* and *M. sinensis* back together). Thus, for *M. sacchariflorus* and *M. sinensis*, speciation seems to be an ongoing, lengthy, and dynamic process, rather than a single discrete event.

Adaptive advantages of interspecific tetraploids to a warming climate provide a possible explanation for the absence of diploid *M. sacchariflorus* in Japan. Temperatures and flora in much of Japan around the last glacial maximum were similar to those of contemporary inland eastern Russia, where diploid *M. sacchariflorus* is common but *M. sinensis* is absent or rare (Winkler and Wang, 1993; Adams and Faure, 1997; Ray and Adams, 2001). When the climate subsequently warmed and *M. sinensis* migrated from a refuge in southeast Asia to Japan ~14 000 years before present (Clark *et al.*, 2014), the cold-adapted *M. sacchariflorus* in Japan may have benefited from the introgression of *M. sinensis* genes that conferred adaptation to warmer environments. Moreover, introgression would have preferentially favoured fitness of a tetraploid *M. sacchariflorus*–*M.*×*giganteus* complex over diploid *M. sacchariflorus* and diploid interspecific hybrids, owing to the tetraploid hybrids’ competitive rhizomatous habit combined with heterosis and adaptation to a warming climate, thus possibly explaining why diploid *M. sacchariflorus* is absent or exceedingly rare in Japan today. This hypothesis is consistent with the prediction of Stebbins (1971) that cross-ploidy introgressions have played a large role in ecological adaptation. Japan’s maritime climate, lacking extreme temperatures, may have driven the *M. sacchariflorus* conversion from diploid to tetraploid more completely than in mainland Asia. Observations from a field trial located in southern Illinois at the Dixon Springs Experiment Station (37.4° N; USDA hardiness zone 6/7) indicate that some diploid *M. sacchariflorus* are unadapted to warm temperate environments (e.g. by flowering and going dormant many months before the end of the growing season), whereas all tested tetraploid *M. sacchariflorus* from Japan are well-adapted to such environments. However, the range of putatively diploid *M. sacchariflorus* in China extends as far south as 28°N (Sacks *et al.*, 2013). As subsequent analyses of population structure allow the identification of the closest living diploid *M. sacchariflorus* relatives of Japanese tetraploid *M. sacchariflorus*, it will be possible to more fully test this hypothesis by comparing the adaptation of these diploid *M. sacchariflorus* to that of their induced tetraploids, their diploid and tetraploid progeny from crosses with diploid *M. sinensis* from southern Japan, and natural tetraploid *M.*×*giganteus* and *M. sacchariflorus* genotypes. Additionally, if the hypothesis is correct, we expect to see introgression from *M. sinensis* into *M. sacchariflorus* press northward as the climate warms.

The new understanding that most of the tetraploid phenotypically *M. sacchariflorus* in Japan are in fact backcross hybrids between *M. sacchariflorus* and *M. sinensis* with variable degrees of introgression from *M. sinensis* also leads to an interesting question of nomenclature. Hodkinson and Renvoize (2001) defined the hybrid between *M. sacchariflorus* and *M. sinensis* as the nothospecies *M.*×*giganteus*. The International Code of Nomenclature for algae, fungi, and plants (McNeill *et al.*, 2012) further indicates that a nothotaxa includes all filial and backcross individuals that are recognizably derived from the defined parental taxa. Molecular markers have allowed pure *M. sacchariflorus* to be distinguished from F_1_ and backcross hybrids with *M. sinensis*, though phenotypically even F_1_ triploid *M.*×*giganteus* can be difficult to distinguish from tetraploid *M. sacchariflorus*. Thus, many of the tetraploid genotypes in Japan that look like *M. sacchariflorus* phenotypically may be most accurately referred to as *M.*×*giganteus*. Perhaps it would be most accurate to refer to this group in Japan as an *M. sacchariflorus*–*M.*×*giganteus* complex. Nomenclature details aside, researchers should be cognizant of the complex nature of the tetraploid *Miscanthus* populations in Japan.

For the development of biomass cultivars, the new triploid F_1_ *M.*×*giganteus* accessions that were identified here and imported into the USA will be immediately useful in field trials to compare their performance to the current agronomic standard, *M.*×*giganteus* ‘Illinois’. Though *M. sacchariflorus* is the maternal parent of ‘Illinois’ (Hodkinson *et al.*, 2002c), the results here indicate that *M. sinensis* can be the maternal parent with similar probability. Crosses to create new *M.*×*giganteus* can therefore be performed in either direction, and maternal cytoplasmic effects on the performance of new hybrids should be investigated. Also, the degree of introgression from *M. sinensis* should now be taken into account when selecting a parent from the tetraploid *M. sacchariflorus*–*M.*×*giganteus* complex for crosses with diploid *M. sinensis* and evaluating the performance of their progeny. As traits of interest are mapped on the *M. sinensis* and *M. sacchariflorus* genomes in the near future, it is possible that a greater genetic dosage from one parent species or the other will be desired for particular genes or genomic regions, in which case particular tetraploid *M. sacchariflorus*–*M.*×*giganteus* accessions may be selected as parents based on which regions of the *M. sinensis* genome they do or do not possess.

### Introgression of M. sacchariflorus DNA into diploid *M. sinensis*

Although much less frequent than introgression of diploid *M. sinensis* into tetraploid *M. sacchariflorus*, introgression of *M. sacchariflorus* DNA into *M. sinensis* was also observed, particularly in Hokkaido (Figs. 1A, C, E and 2). The *M. sinensis* individual with the greatest amount of *M. sacchariflorus* DNA, EBI-2009-02c, was collected in Rikubetsu, the coldest place in Japan, where *Miscanthus* is rare (T. Yamada, personal observation). Hybridization of *M. sinensis* and *M. sacchariflorus* to produce diploid progeny that could backcross to diploid *M. sinensis* is difficult to explain, given that no endemic diploid *M. sacchariflorus* are known to exist in Japan. Recent importation of diploid *M. sacchariflorus* from China is one possible explanation for the presence of interspecific diploid progeny. Another possibility is the production of highly rare monoploid gametes from tetraploid *M. sacchariflorus* or triploid *M.*×*giganteus*. However, *M.*×*giganteus* from the south is unlikely to have contributed to the ancestry of these diploid *M. sinensis*× *M. sacchariflorus* hybrids, which were found in the north, because the diploid hybrids did not have any ancestry from the S Japan *M. sinensis* group. Moreover, in northern Japan tetraploid *M. sacchariflorus* typically flower much later than *M. sinensis*. Lastly, the possibility must be considered that endemic populations of diploid *M. sacchariflorus* either recently existed in northern Japan and have been lost, or exist currently but have remained undetected, and these diploid *M. sacchariflorus* crossed with *M. sinensis*.

### Spatial genetic structure in *M. sinensis*

Spatial principal components analysis revealed several distinct genetic clines in *M. sinensis*, which reflect different demographic processes and their relative importance in shaping the population structure of *M. sinensis* in Japan. The largest eigenvector by far indicated a cline from south to north (Fig. 3A), which could reflect progressive founder effects as *M. sinensis* migrated from southeast China and colonized Japan (Clark *et al.*, 2014). The largest eigenvector also corresponds to geographic distance from Korea and the Ryukyu islands, with which ongoing genetic exchange is probably taking place. The region to the southwest of the Noto Peninsula was also distinguished by the first eigenvector, perhaps because this maritime region is isolated by mountains and ocean from other nearby maritime regions. Consistent with the Structure analysis identifying a distinct central Japan *M. sinensis* group (Fig. 1C), the second eigenvector distinguished central Honshu from northern and southern Japan (Fig. 3B). The Japanese Alps have been a barrier to gene flow, as indicated by a steep cline in this region for the second eigenvector (Fig. 3B) and by greater genetic differentiation for the S Japan group than the other Japanese *M. sinensis* groups to the north of these mountains (Table 2). The combination of the first and second eigenvectors gave a similar pattern to a genetic cline previously found across southern Japan and Korea in *M. sinensis* (Slavov *et al.*, 2013) and is also similar to the pattern that was seen with Structure (Fig. 1A). The third eigenvector showed a cline east to west in central and southern Japan (Fig. 3C), suggesting that there may be gene flow along the coasts that bypasses the clines seen in the first two eigenvectors. Most of the genetic variation between sites (83.2%) remained unexplained by these three eigenvectors, indicating that obligate outcrossing and wind dispersal of seed and pollen, in combination with the relatively recent colonization of Japan by *M. sinensis* (within the past ~14,000 years; Clark *et al.*, 2014) resulted in an unstructured pattern of allele frequencies at most loci.

The plastid results were consistent with those of previous studies (Shimono *et al.*, 2013; Clark *et al.*, 2014), which indicated the presence of two major groups of plastid haplotypes in Japan that probably correspond to two or more colonization events. Additionally, it was found that the eight most common haplotypes for *M. sinensis* in Japan (four from each of the two major groups) all had well-defined geographic ranges (Fig. 4), indicating strong barriers to seed flow. The Tsugaru Strait is one such barrier, given that the Hokkaido population almost exclusively had haplotype C, despite haplotypes H and J being common nearby in northern Honshu. Kyushu is similarly isolated; the only major haplotypes found there are C and I, despite haplotype B being common nearby in southern Honshu and Shikoku. The Japanese Alps also seem to block seed flow, given that haplotypes A, H, J, M, and N were only found north of the mountain range, whereas haplotypes B and I were only found south of it. Cytoplasmic markers can exhibit stronger population structure than nuclear markers owing to undergoing a higher rate of genetic drift as a result of having a smaller effective population size (McCauley, 1995).

### Origins of ornamental cultivars and naturalized *M. sinensis* in the USA

In this study, geographic resolution was added to the previous finding that ornamental and naturalized *M. sinensis* in the USA originated from southern Japan (Clark *et al.*, 2014). The Q values (Fig. 1B) and the geographic distribution of plastid haplotypes (Fig. 4) for the ornamental cultivars indicated that there were multiple introductions to the USA but from two small areas (Fig. 4) of east-central Japan (eastern parts of Kantō and Chūbu) and south-western Japan (Chūgoku and western Kansai). The Q values among the naturalized populations in the USA were less varied than Q values among ornamental cultivars, indicating that these populations originated from a small subset of ornamental cultivars. These findings on the origins of the ornamental cultivars in the USA and Europe are consistent with historical documentation that the Yokohama Nursery Company played an important role in distributing Japanese plants, including *Miscanthus*, internationally during the late 1800s and early 1900s (Galloway, 1907, see entry 10524; http://www.nal.usda.gov/exhibits/speccoll/exhibits/show/nursery-and-seed-trade-catalog/japanese-nursery-and-seed-trad, last accessed 5 January 2015; http://www.yokohamaueki.co.jp/ayumi/index.html, last accessed 5 January 2015). The ornamental *M. sinensis*×*M. sacchariflorus* ‘Purpurascens’ was a likely ancestor of many of the other ornamental cultivars, given that its *M. sinensis* ancestry is from the Korea/N China genetic group (red, Fig. 1A, B), and that ornamental cultivars with ancestry from the Korea/N China cluster tended to have a similar amount of ancestry from *M. sacchariflorus* (Fig. 1B). Among ornamentals and US naturalized accessions with negligible *M. sacchariflorus* ancestry, a small but significant amount of Korea/N China ancestry was present in some genotypes, possibly indicating purifying selection to remove *M. sacchariflorus* genomic regions, given that this pattern of admixture was rare in the native range.

## Conclusions

In Japan, speciation between tetraploid *M. sacchariflorus* and diploid *M. sinensis* is an ongoing and dynamic process, with gene exchange occurring in both directions but asymmetrically in favour of diploid to tetraploid. Tetraploidy seems to have promoted introgression of genes from diploid *M. sinensis* into tetraploid *M. sacchariflorus* in Japan to a greater extent than gene exchange between sympatric diploid *M. sinensis* and diploid *M. sacchariflorus* populations in China. These conclusions are consistent with the theory of Stebbins (1971) that unilateral introgressive hybridization across ploidy levels can play an important role in plant evolution. The *M.*×*giganteus*–*M. sacchariflorus* complex in Japan is expected to be an outstanding resource for developing new biomass cultivars.

To develop improved biomass cultivars of *Miscanthus*, it will be desirable to genetically map agronomic traits in *M. sinensis* and tetraploid *M. sacchariflorus*, and identify the best parents of each species for breeding new triploid *M.*×*giganteus* cultivars. Artificial backcrossed populations, derived from crosses between tetraploid *M.*×*giganteus* and tetraploid *M. sacchariflorus*, will be useful for elucidating the role of *M. sinensis* genes introgressed into a tetraploid *M. sacchariflorus* genetic background. Experiments with such introgressants will provide insights into their possible selective advantage to wild populations of *M. sacchariflorus*, as well as their potential utility for breeding biomass cultivars. Additionally, an understanding of how the degree of *M. sinensis* introgression in tetraploid *M. sacchariflorus*–*M.*×*giganteus* complex genotypes affects heterosis of triploid *M.*×*giganteus* hybrids would be useful for breeding bioenergy cultivars. The *Miscanthus* cultivars that are ultimately developed for the bioenergy industry are likely to be complex hybrids possessing traits from multiple species and geographic regions.

## Supplementary data

Supplementary data are available at *JXB* online.

Figure S1. Choice of K in Structure analysis.

Figure S2. Validation of Structure for detecting introgression.

Figure S3. Screeplot from sPCA.

Table S1. Expected and observed Q values from Structure runs on simulated hybrid individuals.

Supplementary Dataset S1. A Microsoft Excel file containing a table of collection data and genetic results by individual, as well as a table providing the sequences of 289 pairs of RAD-tags that are highly diagnostic of *M. sinensis* vs *M. sacchariflorus*.

Supplementary Data
