# Supplementary material for: Genetic structure of Miscanthus sinensis and Miscanthus sacchariflorus in Japan indicates a gradient of bidirectional but asymmetric introgression
Source: J Exp Bot. 2015 Jan 24;66(14):4213–25. doi: 10.1093/jxb/eru511 (PMC4493777; doi:10.1093/jxb/eru511)
Supplement: Supplementary Data [file supp_66_14_4213__index.html]

Genetic structure of Miscanthus sinensis and Miscanthus sacchariflorus in Japan indicates a gradient of bidirectional but asymmetric introgression — Genetic structure of Miscanthus sinensis and Miscanthus sacchariflorus in Japan indicates a gradient of bidirectional but asymmetric introgression — Supplementary Data 

# Genetic structure of *Miscanthus sinensis* and *Miscanthus sacchariflorus* in Japan indicates a gradient of bidirectional but asymmetric introgression

## Supplementary Data

Data files

**Files in this Data Supplement:**

- Supplementary Data - Supplementary Data
- Supplementary Data - Supplementary Data
