# Supplementary material for: Genetic structure of Miscanthus sinensis and Miscanthus sacchariflorus in Japan indicates a gradient of bidirectional but asymmetric introgression
Source: J Exp Bot. 2015 Jan 24;66(14):4213–25. doi: 10.1093/jxb/eru511 (PMC4493777; doi:10.1093/jxb/eru511)
Supplement: Supplementary Data [file supp_eru511_jexbot135939_file001.pdf]

Genetic structure of *Miscanthus sinensis* and *M. sacchariflorus* in Japan indicates a gradient of bidirectional but asymmetric introgression

Lindsay V. Clark, J. Ryan Stewart, Aya Nishiwaki, Yo Toma, Jens Bonderup Kjeldsen, Uffe Jørgensen, Hua Zhao, Junhua Peng, Ji Hye Yoo, Kweon Heo, Chang Yeon Yu, Toshihiko Yamada, and Erik J. Sacks

Supplementary Materials and Methods

Plant materials and genotyping

Seeds were germinated under aseptic conditions at the University of Illinois, and plants derived from seed were maintained in a greenhouse, with the exception of 15 accessions collected in 1996 that were germinated and maintained in a field at Aarhus University at Foulum, Denmark.

Freeze-dried leaf tissue was pulverized in a Mini Beadbeater (Biospec Products, Bartlesville, Oklahoma, USA) or GenoGrinder (SPEX, Metuchen, New Jersey, USA), then used for DNA extraction with a modified CTAB method.

Structure analysis

SNPs were analyzed with the software Structure 2.3.4 (Falush *et al.*, 2003) to identify new genetic groups, assign individuals to previously identified groups (Clark *et al.*, 2014), and detect admixture and hybridization between species. Structure Harvester (Earl and VonHoldt, 2011) was used to determine the best value of K. To determine the origins of ornamental and naturalized accessions of *M. sinensis* available in the US, the USEPOPINFO and PFROMPOPFLAGONLY options were used. To detect population structure in the Japanese collections, a set of Structure runs was performed using only individuals from Japan, excluding the Ryukyu Islands, (379 individuals per run; 253 from the Japan dense-sampling set, minus two that were identified as non-native to Japan in previous Structure runs, plus 128 from the region-wide set) with USEPOPINFO = FALSE at K = 1 through 10. To determine the relationship between *Miscanthus* from Japan and those in mainland Asia, a set of Structure runs was performed that included the 645 genotypes in the region-wide set (Clark *et al.*, 2014) in addition to the Japan dense-sampling set. To avoid biased clustering due to multiple individuals coming from one seed accession (which in many cases represented family groups) five different Structure input files were generated, each including one individual from each of 253 accessions in the Japan dense-sampling set. Structure results indicated that two accessions were a mix of *M. sacchariflorus* and *M. sinensis*; these two accessions were then split, resulting in the 255 accessions listed in Table 1. For accessions with multiple individuals, each individual was assigned at random to at least one Structure input file. In combination with the region-wide set, each Structure input file therefore included genotypes from 898 individuals. Each input file was

run for one iteration of K = 1 through 10. The burnin consisted of 10,000 MCMC reps, followed by 50,000 MCMC reps after the burnin. Other parameters were left at defaults. Data were processed in a diploid format; Wang *et al*. (2013) demonstrated that introgression in polyploids can be quantified using RAD-seq data without knowledge of allele copy number.

To determine the origins of ornamental and US naturalized collections, a second set of Structure runs was performed that included the above individuals, plus 81 ornamental cultivars and 42 naturalized individuals from the USA. For this set, USEPOPINFO and PFROMPOPFLAGONLY were set to TRUE, MIGPRIOR was set to 0.05 (to reflect the large amount of admixture between populations) and GENSBACK was set to 4 (with the expectation that RAD-seq SNPs would be sensitive enough to detect BC_3 individuals). The POPFLAG column was set to 1 for native individuals and 0 for ornamental and naturalized individuals (Fig. 1B). Eight native populations were delimited in the POPINFO column using population assignments made with Discriminant Analysis of Principal Components (DAPC; Jombart *et al*., 2010), which were similar to the population assignments made with Structure at K=8 (Fig. 1A).

The validity of our Structure results for detecting introgression between species was confirmed using two independent methods: principal component analysis (PCA) of the same 1513 individuals that were evaluated by Structure, and Structure analysis of 1100 simulated hybrid and backcross individuals and 100 simulated individuals representing the common ancestor of *M. sinensis* and *M. sacchariflorus*. For PCA, we filtered our SNP dataset, only retaining 3490 SNPs with less than 5% missing data; filtering prevented individuals with high missing data rates from appearing as hybrid individuals due to missing data imputation. The *glPca* function from adegenet (Jombart and Ahmed, 2011) with *scale=TRUE* was then used to perform PCA. A linear model, with the value on the first principal component axis as the dependent variable, and the Structure Q value representing proportion *M. sacchariflorus* ancestry as the independent value, was fitted using R 3.1.1 (Supplementary Figure 2A).

In order to simulate hybrid and ancestral individuals for the second method to validate Structure results, "pure" individuals were first selected from the dataset, with the criteria that they had to have a Structure Q value greater than 0.95 for the *M. sacchariflorus* cluster (36 individuals from Japan and nine from China) or the sum of the three Japanese *M. sinensis* clusters (752 individuals from Japan). The SNP set was reduced to the 19,124 loci that did not have missing data in all pure *M. sacchariflorus* individuals. Missing data were imputed as the median genotype within the two sets of pure individuals, which were then sampled randomly to be used as parents to generate simulated hybrids, including 100 individuals each of F1 and BC1 through BC5 (1100 individuals total), with separate backcross classes depending on which species was the recurrent parent. In order to simulate individuals from an ancestral population from which *M. sinensis* and *M. sacchariflorus* diverged, the major (most common) allele was identified at each locus for the 45 pure *M. sacchariflorus* and 1105 pure *M. sinensis* (including 752 from Japan and 353 from mainland Asia). At the 16,096 loci at which *M. sacchariflorus* and *M. sinensis* had the same major allele, all 100 simulated ancestral individuals were homozygous for

the major allele. At the 3028 loci at which *M. sinensis* and *M. sacchariflorus* had different major alleles, genotypes were simulated assuming that the two alleles were at equal frequency in the ancestral population. All simulated individuals had missing data inserted randomly at a rate of 22% to match the missing data rate in the "pure" populations. An input file was then generated for Structure, including genotypes of 1150 pure *M. sinensis* and *M. sacchariflorus* and 1200 simulated individuals at 19,124 loci. Five structure runs were performed at K=8 using a burnin of 10,000 MCMC reps followed by 50,000 MCMC reps after burnin, under default conditions as used in the analysis of native *Miscanthus*. Results are presented in Supplementary Figure 2B and Table S1.

Spatial Principal Components Analysis

Spatial principal components analysis (sPCA), implemented in the R package adegenet (Jombart *et al.*, 2008) was used to identify spatial patterns in genetic variation of *M. sinensis* across the major islands of Japan using RAD-seq SNPs. This technique transforms molecular marker data into eigenvectors similarly to principal component analysis, but maximizes the product of spatial autocorrelation and variance of each eigenvector rather than maximizing the variance alone. Latitude and longitude of each collection site were rounded to two decimal digits to lump individuals into 205 collection sites, which included 198 *M. sinensis* accessions (654 individuals, excluding two accessions with no collection site data, two accessions that were identified in Structure analysis as having non-Japanese origin, and one individual with >20% ancestry from *M. sacchariflorus* according to Structure) from the Japan dense-sampling set and 128 individuals of Japanese origin from the region-wide set. Allele frequencies were estimated for each collection site. Out of the 20,704 RAD-seq SNPs, 19,148 were variable within this dataset. All SNPs that were variable in *M. sinensis* with missing data at four or fewer collection sites were retained for sPCA analysis (5,359 SNPs). A Gabriel graph was generated to represent geographic connections between collection sites. The number of eigenvectors to interpret was chosen based upon examination of a screeplot of eigenvalues (Fig. S3). The lag vector of each interpreted eigenvector was plotted against latitude and longitude using the *s.image* function from the R package ade4 (Chessel *et al.*, 2004) with the argument *span=0.4* to control the degree of smoothing.

Flow cytometry

Approximately 1 cm^2 of leaf tissue from *Miscanthus* samples was co-chopped with *Sorghum bicolor* 'Nr481' (IPK Gatersleben, Germany) as an internal standard for 3-5 min in extraction buffer on ice. Chopped samples were mixed with 10 ml extraction buffer and filtered through a 50 μm mesh, then centrifuged at 2100 rpm for 20 min. Nuclei were then stained with propidium iodide as previously described (Rayburn *et al.*, 2009) and run on an LSR II Flow Cytometry Analyzer (BD Biosciences, San Jose, California, USA) at the Roy J. Carver Biotechnology Center at the University of Illinois. DNA content was determined based on median G1 peak area, assuming 1.74 pg/2C for sorghum.

References

Chessel D, Dufour AB, Thioulouse J. 2004. The ade4 package - I : One-table methods. R News **4**, 5–10.

Clark L V, Brummer JE, Głowacka K, *et al.* 2014. A footprint of past climate change on the diversity and population structure of *Miscanthus sinensis*. Annals of Botany **114**, 97–107.

Earl DA, VonHoldt BM. 2011. STRUCTURE HARVESTER: a website and program for visualizing STRUCTURE output and implementing the Evanno method. Conservation Genetics Resources **4**, 359–361.

Falush D, Stephens M, Pritchard JK. 2003. Inference of population structure using multilocus genotype data: Linked loci and correlated allele frequencies. Genetics **164**, 1567–1587.

Jombart T, Ahmed I. 2011. adegenet 1.3-1: new tools for the analysis of genome-wide SNP data. Bioinformatics **27**, 3070–3071.

Jombart T, Devillard S, Balloux F. 2010. Discriminant analysis of principal components: a new method for the analysis of genetically structured populations. BMC Genetics **11**, 94.

Jombart T, Devillard S, Dufour A-B, Pontier D. 2008. Revealing cryptic spatial patterns in genetic variability by a new multivariate method. Heredity **101**, 92–103.

Rayburn AL, Crawford J, Rayburn CM, Juvik JA. 2009. Genome size of three *Miscanthus* species. Plant Molecular Biology Reporter **27**, 184–188.

Wang N, Thomson M, Bodles WJA, Crawford RMM, Hunt H V, Featherstone AW, Pellicer J, Buggs RJA. 2013. Genome sequence of dwarf birch (*Betula nana*) and cross-species RAD markers. Molecular Ecology **22**, 3098–111.

Supplementary Figures

Supplementary Fig. S1. Choice of K in Structure and DAPC analysis. A) DeltaK plot for the East Asia-wide set combined with the Japan dense-sampling set. B) Delta K plot for Japan only. C-D) Barplots of Q values, sorted by Q, for five Structure runs on Japan only, with C) K=4 and D) K=5. Colors correspond to those used in the main manuscript. E) Baysian Information Criterion (BIC) plot from DAPC analysis, using the East Asia-wide set combined with the Japanese dense-sampling set.

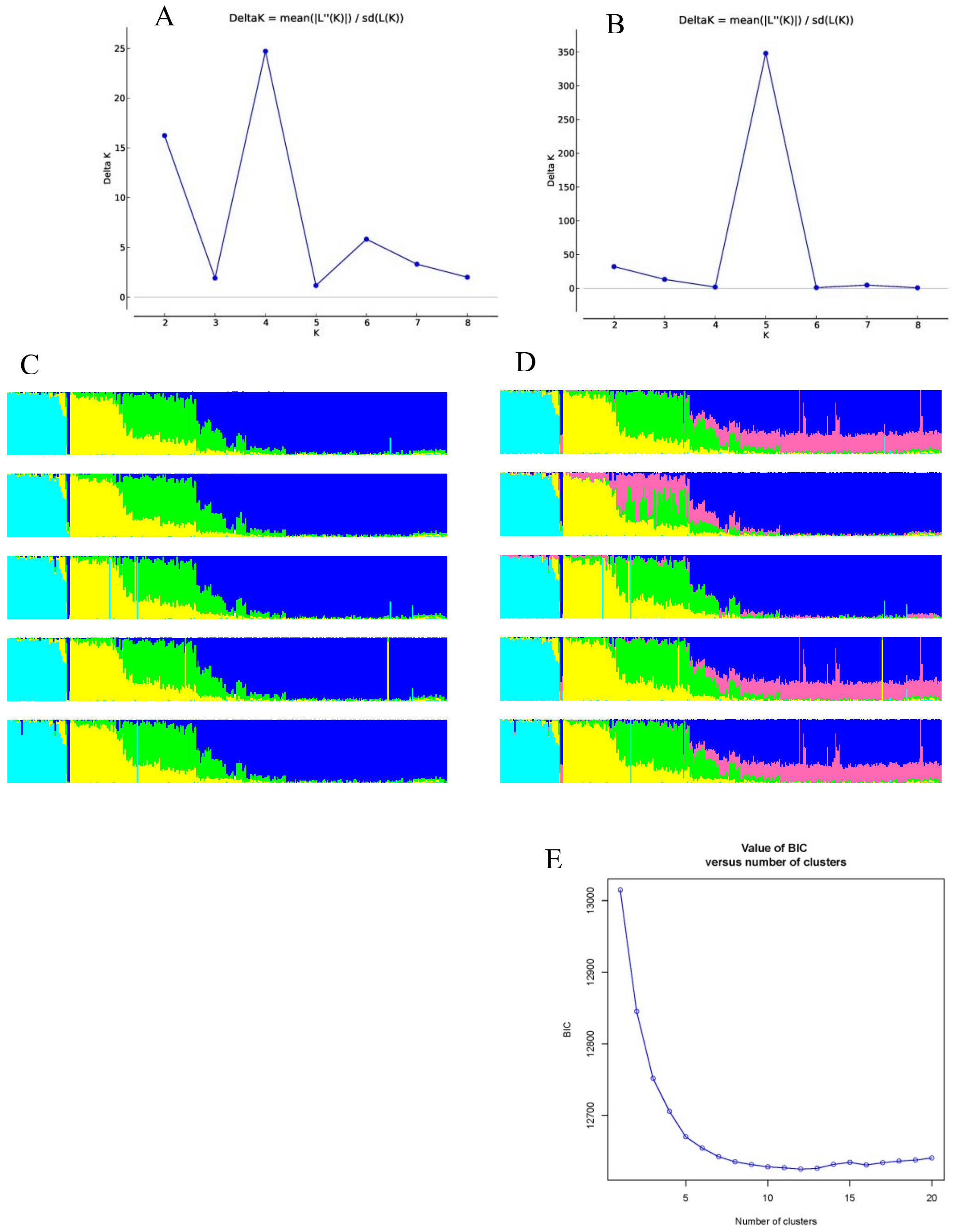

Supplementary Fig. S2. Validation of Structure for detecting hybridization and introgression between *M. sinensis* (Msi) and *M. sacchariflorus* (Msa). A) Linear model fitting the first principal component of 3490 SNPs with less than 5% missing data to the proportion of Msa ancestry according to Structure analysis using 20,704 SNPs. Results from 1513 individuals were used in the model, each represented as one point in the graph. B) Barplots of Q values from five Structure runs on 1105 pure Msi individuals, 45 pure Msa individuals, 1100 simulated hybrids, and 100 simulated ancestors of Msi and Msa. Msi from Japan only were used as parents of simulated hybrids with Msa.

A

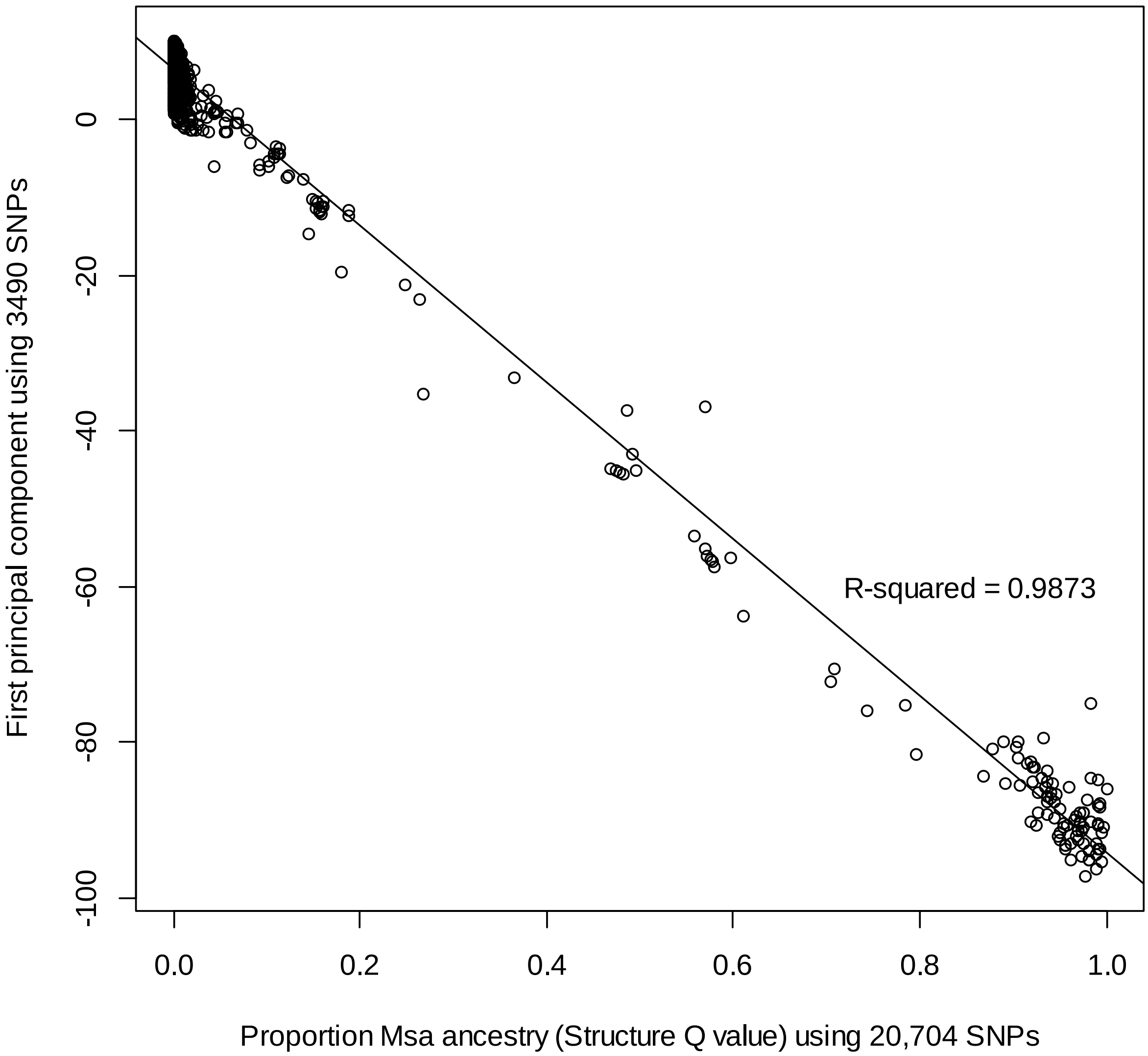

B

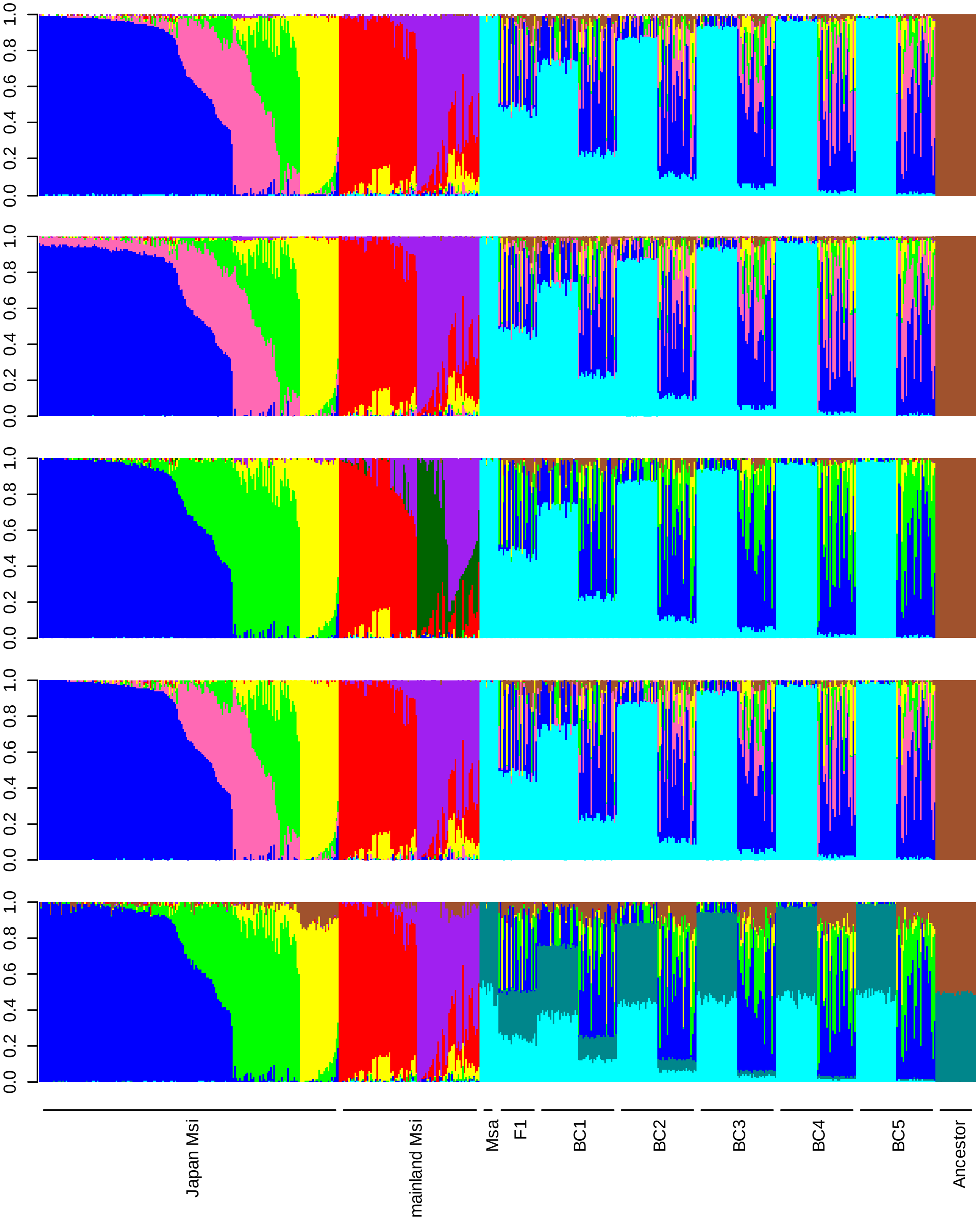

0.0
0.2
0.4
0.6
0.8
1.0
Japan Msi
mainland Msi
Msa
F1
BC1
BC2
BC3
BC4
BC5
Ancestor

Supplementary Fig. S3.   Screeplot from spatial principal components analysis. The three eigenvectors that were retained in Fig. 3A, B, and C are indicated as V1, V2, and V3, respectively.

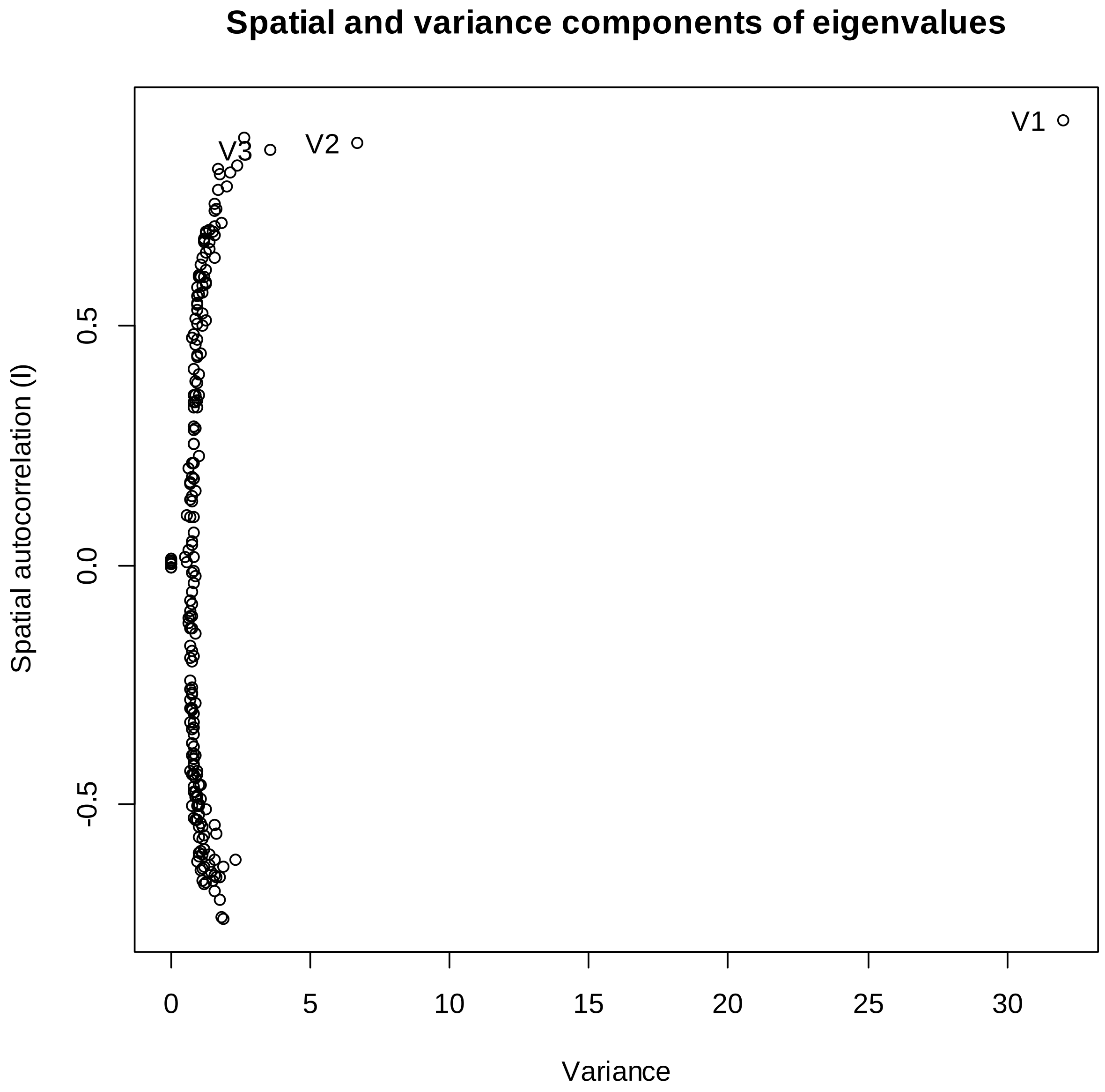

Supplementary Table S1. Expectations, means, and standard deviations (SD) of Q values indicating the proportion of *M. sacchariflorus* ancestry, according to Structure analysis of 19,124 SNPs, of non-admixed ("pure") *M. sinensis* (Msi) and *M. sacchariflorus* (Msa) individuals and simulated hybrids. The number of individuals examined is 1105 for pure Msi, 45 for pure Msa, and 100 for each hybrid class in the table. For backcross (BC) classes, the recurrent parent species is indicated.

	Expected Q	Mean Q (Structure)	SD of Q (Structure)
Pure Msi	0.000	0.002	0.004
BC5 Msi	0.016	0.012	0.006
BC4 Msi	0.031	0.026	0.007
BC3 Msi	0.063	0.055	0.010
BC2 Msi	0.125	0.111	0.015
BC1 Msi	0.250	0.233	0.022
F1	0.500	0.484	0.024
BC1 Msa	0.750	0.743	0.018
BC2 Msa	0.875	0.874	0.012
BC3 Msa	0.938	0.939	0.008
BC4 Msa	0.969	0.972	0.006
BC5 Msa	0.984	0.987	0.004
Pure Msa	1.000	0.992	0.010
